# Population pharmacokinetics of caspofungin in critically ill Chinese children: a prospective observational study

**DOI:** 10.1128/aac.01277-25

**Published:** 2025-12-30

**Authors:** Nuo Xu, Yufei Shi, Gehang Ju, Xin Liu, Gangfeng Yan, Yi Zheng, Shuyu Hou, Xiaoqiang Xiang, Guoping Lu, Dongsheng Ouyang, Xiao Zhu, Yixue Wang

**Affiliations:** 1 Department of Clinical Pharmacy and Pharmacy Administration, School of Pharmacy, Fudan University 12478, Shanghai, China; 2 School of Pharmacy, Faculty of Medicine, The Chinese University of Hong Kong 26451, Hong Kong SAR, China; 3 Department of Clinical Pharmacology, Xiangya Hospital, Central South University 618619 , Changsha, China; 4 Pediatric Intensive Care Unit, Children's Hospital of Fudan University, National Children's Medical Center 608785 , Shanghai, China; 5 DMPK department, Suzhou Frontage New Drug Development Co.,Ltd, Suzhou, Jiangsu, China; 6 State Key Laboratory of Advanced Drug Formulations for Overcoming Delivery Barriers, Fudan University 12478, Shanghai, China; 7 Department of Pediatrics, Huashan Hospital of Fudan University 12478, Shanghai, China; University Children’s Hospital Münster, Münster, Germany

**Keywords:** caspofungin, ECMO, pediatrics, population pharmacokinetics, intensive care units

## Abstract

While caspofungin is increasingly used to treat invasive fungal infections in pediatric intensive care unit (PICU) patients, its pharmacokinetic profile in this population remains poorly understood, and current dosing regimens are not firmly supported by scientific evidence. This study aimed to characterize the population pharmacokinetics of caspofungin in critically ill children and to identify dosing strategies for optimal exposure. The prospective clinical study was conducted among pediatrics in PICU. Population pharmacokinetic analysis and Monte Carlo simulations were performed. A total of 138 plasma samples collected from 29 pediatric patients (0.33–16 years) were included in the final analysis. The two-compartment model with allometric scaling on body surface area (BSA, exponential 1 for volume of distribution and 0.66 for clearance) accurately described time courses of caspofungin. Extracorporeal membrane oxygenation (ECMO) significantly increased the central volume of distribution (effect coefficient 18.2). There was no significant difference in area under the concentration curve (AUC) between patients with and without ECMO support. Simulations demonstrated that tAUC_ss,24h_/MIC-based PTA results showed no significant differences between ECMO and non-ECMO groups and supported the current dosing regimen. A fixed maintenance dose (MD) is appropriate for patients with BSA ≥ 1.4 m², while the standard BSA-based MD remains preferable for those with BSA <1.4 m². Our study confirmed the recommended caspofungin dosing regimen in Chinese critically ill PICU patients. Although the number of patients receiving ECMO in this study was limited, future studies with a larger ECMO population are warranted to further validate these findings.

This study is registered with ClinicalTrials.gov as NCT04961593.

## INTRODUCTION

Over the past two decades, the incidence of invasive fungal infections (IFIs) has steadily increased. IFIs have become a prevalent hospital-acquired infection, 50% of which occur in patients in the pediatric intensive care unit (PICU) (1, 2). Caspofungin, the first echinocandin approved for treating IFIs, is effective against infections caused by *Candida* and *Aspergillus* species. However, the pathophysiological status of children in PICU, particularly those with hypoalbuminemia, liver dysfunction, could alter the metabolism of caspofungin (3). Pharmacokinetic (PK) variabilities and suboptimal drug exposure with standard dosing, especially in critically ill pediatric patients, result in unpredictable drug behavior and an increased risk of therapeutic failure (4). Despite these challenges, comprehensive PK studies on caspofungin in this vulnerable population remain limited, underscoring the need for further research in this area (5).

Additionally, extracorporeal membrane oxygenation (ECMO), a common ICU intervention, can be life-saving but is associated with an increased risk of nosocomial infections, particularly fungal infections like *Candida*, which can worsen patient outcomes (6). Studies have shown that ECMO circuits and priming solutions could contribute to drug adsorption, potentially increasing the volume of distribution of certain medications (7, 8). One study in adult ECMO patients suggested a higher dose for those with a lean body weight (LBW) of 40–60 kg, while another study found no significant differences in PKs between ECMO and non-ECMO patients. However, PK studies of caspofungin in ECMO patients have primarily focused on adults, with similar studies lacking in pediatric intensive care unit (PICU) children (9). Given this limitation, although relevant pharmacokinetic studies exist in adults, children differ markedly in organ development, body fluid composition, and drug metabolism. Under critical illness condition or ECMO, these differences may be further amplified due to higher circuit-to-blood volume ratio and greater drug adsorption to circuit surfaces, making direct extrapolation of adult ECMO PK data to children inappropriate. This highlights a critical gap in PK research and raises concerns about the adequacy of current dosing guidelines for this vulnerable population.

However, conducting clinical PK studies in pediatrics poses several significant challenges. Ethical guidelines are stricter than for adults, requiring minimal sampling and distress (10). Children are a vulnerable population, and caregivers may be reluctant to consent (11). Limited blood volume and discomfort make sample collection difficult, while financial and staffing demands further complicate such studies (12). Optimal design theory enables researchers to use mathematical optimization algorithms to structure data collection in a way that best addresses specific research questions (13).

This study aimed to characterize the PKs of caspofungin in critically ill pediatric patients and to identify sources of PK variability among those receiving ECMO support. Based on the developed population pharmacokinetic (PopPK) model, we subsequently evaluated dosing regimens to improve therapeutic efficacy in PICU children, with or without ECMO support.

## MATERIALS AND METHODS

### Study design

A prospective clinical study was conducted among pediatrics in PICU from Children’s Hospital of Fudan University from 1 November 2022 to 30 December 2024. Children aged 3 months to 18 years who were admitted to PICU and received caspofungin therapy were eligible for inclusion. The study population encompassed critically ill children, including but not limited to those with liver insufficiency, hypoproteinemia, ECMO treatment, continuous renal replacement therapy (CRRT), or sepsis. Eligible subjects were administered a once-daily, 1-h intravenous infusion of caspofungin, following a body surface area (BSA)-based dosage regimen: a loading dose (LD) of 70 mg/m² (not to exceed 70 mg) on the first day, followed by 50 mg/m² (not exceed 70 mg) for maintenance dose (MD). BSA was calculated using the Mosteller formula: $BSA(m^{2})=\sqrt{\frac{Weight(kg)*Height(cm)}{3600}}$ (14). All participants were provided written consent prior to participating in the study (US Clinical Trials, NCT04961593). Demographic and baseline clinical laboratory data were collected from the medical records, including age, sex, body weight, height, BSA, liver function, renal function, and various biomarkers reflecting immune system status. Information on bacterial cultures was also collected from microbiological laboratory reports. The biochemical measurements were derived from the most recent clinical laboratory results available within 7 days prior to the first caspofungin administration, reflecting the patient’s baseline physiological status.

### Caspofungin assay and fungal cultures

Total caspofungin plasma concentrations were quantified using a liquid chromatography tandem mass spectrometry (LC-MS/MS) method. Blood samples were collected with EDTA-K2 as anticoagulant, and plasma was obtained by centrifugation at 4,000 *g* for 5 min and stored at −80°C until analysis, which would be performed within 30 days. The analysis was performed using the LC-40AD chromatographic system (Shimazu, Tokyo, Japan) combined with a 6500+ Triple Quad mass spectrometry system (AB Sciex, Framingham, USA). The separation is achieved on a Kinetex C18 column (2.1 × 50 mm, 2.6 μm) using a mobile phase composed of buffer (0.1% [vol/vol] formic acid and 5 mM ammonium acetate) and methanol (0.1% [vol/vol] formic acid). A volume of 40 μL of each plasma sample was precipitated with 260 μL of methanol with 0.4% formic acid. After centrifugation, 150 μL of the supernatants was mixed with 150 μL of water, and a volume of 1 μL of mixture was injected into the chromatographic system. The calibration range of caspofungin was linear over the range of 0.05 to 50 μg/mL with a lower limit of quantification (LLOQ) of 0.05 μg/mL. The samples with concentration that exceeded the upper limit were diluted into half by using blank plasma sample. All the analysis run met the requirement of M10 guideline for bioanalytical method validation and study sample analysis (15).

Central venous blood samples were collected for fungal culture as part of routine microbiological testing in the microbiology laboratory of Children’s Hospital of Fudan University (Shanghai, China). Fungal isolates were identified using matrix-assisted laser desorption/ionization time-of-flight mass spectrometry (MALDI-TOF MS). Antifungal susceptibility testing, including determination of the minimum inhibitory concentration (MIC) for caspofungin, was performed using the broth microdilution method in accordance with the Clinical and Laboratory Standards Institute (CLSI) M27-A4 guidelines. The MIC breakpoints were interpreted according to CLSI criteria.

### Sampling design

This study incorporated data from two study stages: the intensive sampling stage, followed by a sparse sampling stage (Fig. S1). In the first stage, the sampling time points were scheduled at pre-dose, 1, 2, 4, 8 h (if feasible) and 16 h (if feasible) following the most recent dose (the sixth administration after the first dose). The optimal sparse sampling design was then conducted to minimize the number of sampling points based on the defined constraint conditions. The designed sparse sampling time windows were pre-dose (0–1 h), 0.5–1.5, 6–7, and 23–24 h after the prior dose (the sixth administration after the first dose). The detailed optimal design process was described in the Supplemental material.

Design software tools, including $DESIGN option in NONMEM (version 7.5, ICON Development Solutions, Ellicott City, Maryland) and PopED (version 0.6.0, R package), were utilized. These tools evaluated uncertainties in PK parameter estimates based on the current sampling scheme and PopPK model. The designs were evaluated to ensure the precise estimation of typical PK parameters, defined as a relative standard error (RSE) of less than 50%.

### Population pharmacokinetic modeling of caspofungin

The PopPK model was developed using the non-linear mixed-effects modeling software NONMEM (version 7.5, ICON Development Solutions, Ellicott City, Maryland) with the first-order conditional estimation with interaction (FOCE-I) algorithm. If the proportion of data below the quantification limit (BQL) was less than 10%, these data were excluded from the modeling process (M1) (16). Considering pediatric physiological features, allometric scaling for body size metrics, including weight, BSA, LBW, and fat-free mass (FFM) was employed *a priori* (17, 18). The inter-individual variability (IIV) was described by an exponential model, while the residual error for caspofungin concentration was tested by proportional error, additive error, and combined error model.

Subsequently, demographic information (e.g., age, sex), laboratory results (e.g., white blood cell [WBC], red blood cell [RBC], hemoglobin [HB], aspartate aminotransferase [AST], alanine aminotransferase [ALT], total bilirubin [TBIL], serum creatinine [SCR], and uric acid [UA]), and use of ECMO or continuous renal replacement therapy (CRRT) were investigated in stepwise covariate modeling (SCM) (forward selection: *P* < 0.05; backward elimination: *P* < 0.01). The final determination of covariates was based on statistical evidence and clinical insights. The final model was evaluated and determined based on successful convergence, objective function value (OFV), Akaike information criterion (AIC), parameter precision, goodness-of-fit (GoF) plots (19), visual predictive check (VPC) (20) plots, and bootstrap (*n* = 1,000).

To identify clinically relevant covariates on the steady-state 24-h area under concentration-time curve (AUC_ss,24h_) and through concentration (C_min,ss_), the impact of marginal effects changing one covariate at a time on exposure levels was explored using the R package “coveffectsplot.”

### Model-based dose simulation

The final PopPK model was used to perform Monte Carlo simulations (*n* = 1,000) across various dosing regimens to estimate the probability of target attainment (PTA) against different *Candida* species. A PTA of ≥90% was considered indicative of optimal antifungal exposure. Two pharmacokinetic/pharmacodynamic (PK/PD) targets were applied: (i) a total AUC_ss,24h_ to minimal inhibitory concentration (MIC) ratio (tAUC_ss,24h_/MIC) exceeding 450, 865, and 1,185 (associated with a 1−log_10_ kill after 24 h of *C. glabrata*, *C. albicans,* and *C. parapsilosis*, separately) (21) and (ii) a C_min,ss_ above the fixed MIC_90_ (the lowest concentration of caspofungin at which 90% of the isolates were inhibited) of *C. albicans* (0.06 mg/L), *C. glabrata* (0.06 mg/L), and *C. parapsilosis* (1.0 mg/L). Simulations were performed across a range of MIC values.

Virtual populations were generated from the original data set and stratified based on BSA and the use of ECMO support. For patients with BSA ≤ 1.4 m^2^, various MDs, including 10, 20, 30, 40, 50, 60, and 70 mg/m^2^, were simulated. For patients with BSA > 1.4 m^2^, a flat dosing strategy was employed, which encompassed simulated maintenance doses exceeding the standard maximum regimen of 70 mg/day. Given that the study focused on the steady-state PKs of caspofungin, the simulation strategy was accordingly tailored to optimize the MDs.

## RESULTS

### Patients and mycological findings

A total of 29 critically ill pediatric patients were included in this study (Table 1). The median age was 4.63 years (range: 0.33–16 years), and the median body weight was 15.9 kg (range: 4.9–64 kg). Four children received the treatment of ECMO during the administration of caspofungin, and one patient underwent CRRT. Prior to initiating caspofungin, five children (35.7%) showed abnormal liver function tests (22). Additionally, five patients (35.7%) exhibited moderate renal impairment, with an estimated glomerular filtration rate (eGFR) of 30–59 mL/min/1.73 m^2^. Thirteen patients had positive fungal culture reports during caspofungin infusion, and all of them eventually achieved successful culture conversion to negative. The most commonly isolated species was *C. parapsilosis* (*n* = 9), followed by *C. albicans* (*n* = 2), *C. tropicalis* (*n* = 1), and *C. guilliermondii* (*n* = 1).

**TABLE 1:** Demographic and laboratory information of patients^*a*^

Patient characteristics	Median [min, max] (*n* = 29)
ECMO (yes/no)	4/25
CRRT (yes/no)	1/28
Severe malnutrition (yes/no)	2/27
Hypoalbuminemia (yes/no)	2/27
Renal transplant (yes/no)	1/28
Demographics	
Age (years)	5.33 [0.330, 16.0]
Sex (male/female)	12/17
Weight (kg)	16.0 [4.90, 74.0]
Height (cm)	104 [54.0, 173]
BSA (m^2^)	0.660 [0.286, 1.89]
Hematologic parameters	
White blood cell (10^9^/L)	4.92 [0.100, 54.1]
Red blood cell (10^12^/L)	2.98 [1.95, 5.45]
Hemoglobin (g/L)	85.5 [54.0, 168]
Platelet (10^9^/L)	144 [19.0, 588]
Albumin (g/L)	35.8 [24.8, 49.9]
Total protein (g/L)	58.8 [33.3, 75.4]
Hepatic function biomarkers	
Alanine aminotransferase (U/L)	24.1 [3.51, 2,510]
Aspartate aminotransferase (U/L)	50.0 [18.3, 10,900]
Total bilirubin (umol/L)	8.50 [2.10, 329]
Direct bilirubin (umol/L)	4.10 [1.00, 262]
Renal function biomarker	
Serum creatinine (μmol/L)	29.7 [6.50, 223]
Uric acid (μmol/L)	140 [6.69, 736]

Organisms isolated	
*C. parapsilosis*	9
*C. albicans*	2
*C. tropicalis*	1
*C. guilliermondii*	1

^*a*^ ECMO: extracorporeal membrane oxygenation; CRRT: continuous renal replacement therapy; BSA: body surface area.

### Population pharmacokinetics analysis

A total of 138 total plasma caspofungin concentrations (median: 7.475 mg/L, range: 0.155–58.300 mg/L) were collected, all of which were above LLOQ. The final model was a two-compartment model with first-order absorption and first-order elimination. Compared to weight, LBW and FFM, allometric scaling with BSA (scaled to a 0.79 m^2^ individual, a fixed exponential of 1 for volume of distribution [V], and 0.66 for clearance [CL]) yielded the lowest OFV and AIC (Table 2). There was a high correlation between the IIV in CL and the V_1_ (Corr = 0.802); therefore, a correlation coefficient was introduced. SCM identified ECMO as a significant covariate (ΔOFV = −13.262, *P* < 0.001) for the central volume of distribution (V_c_).

**TABLE 2:** Parameter estimates of the final caspofungin population pharmacokinetic model

Fixed parameters	Estimates	RSE^*a*^	Bootstrap (*n* = 1,000) [95% CI^*c*^]
CL, L/h	0.196	17%	0.194 [0.157–0.247]
V1, L	2.22	41%	2.10 [0.58–3.38]
Q, L/h	1.01	57%	0.95 [0.25–3.47]
V2, L	1.63	30%	1.89 [1.20–4.29]
BSA_CL	0.66 FIX	/	/
BSA_V	1 FIX	/	/
ECMO_V_1_	18.2	62%	17.8 [4.00–298.30]

Inter-individual variability (%CV)	Estimates	RSE [SHR]^*b*^	Bootstrap (*n* = 1,000) [95% CI^*c*^]
CL	50.7%	24%/[4%]	47.4% [24.3%–72.7%]
Cor.CL.V_1_	72%	/	54.7% [24.1%–83.3%]
V1	91%	24%/[7%]	92.7% [46.0%–174.1%]

Residual error	Estimates	RSE [SHR]^*b*^	Bootstrap (*n* = 1,000) [95% CI^*c*^]
Prop.error for total concentration, %	17.9%	13%/[17%]	17.9% [11.8%–27.6%]
Add.error for total concentration, mg/L	0.838	29%/[17%]	0.811 [0.180–1.222]

^*a*^ RSE: relative standard error.

^*b*^ SHR: shrinkage.

^*c*^ CI: confidence interval; CL: clearance; V_1_: volume of the central compartment；Q: inter-compartment exchange rate; V_2_: volume of the peripheral volume; BSA: body surface area; ECMO: extracorporeal membrane oxygenation; /, not available.

**Fig 1:**
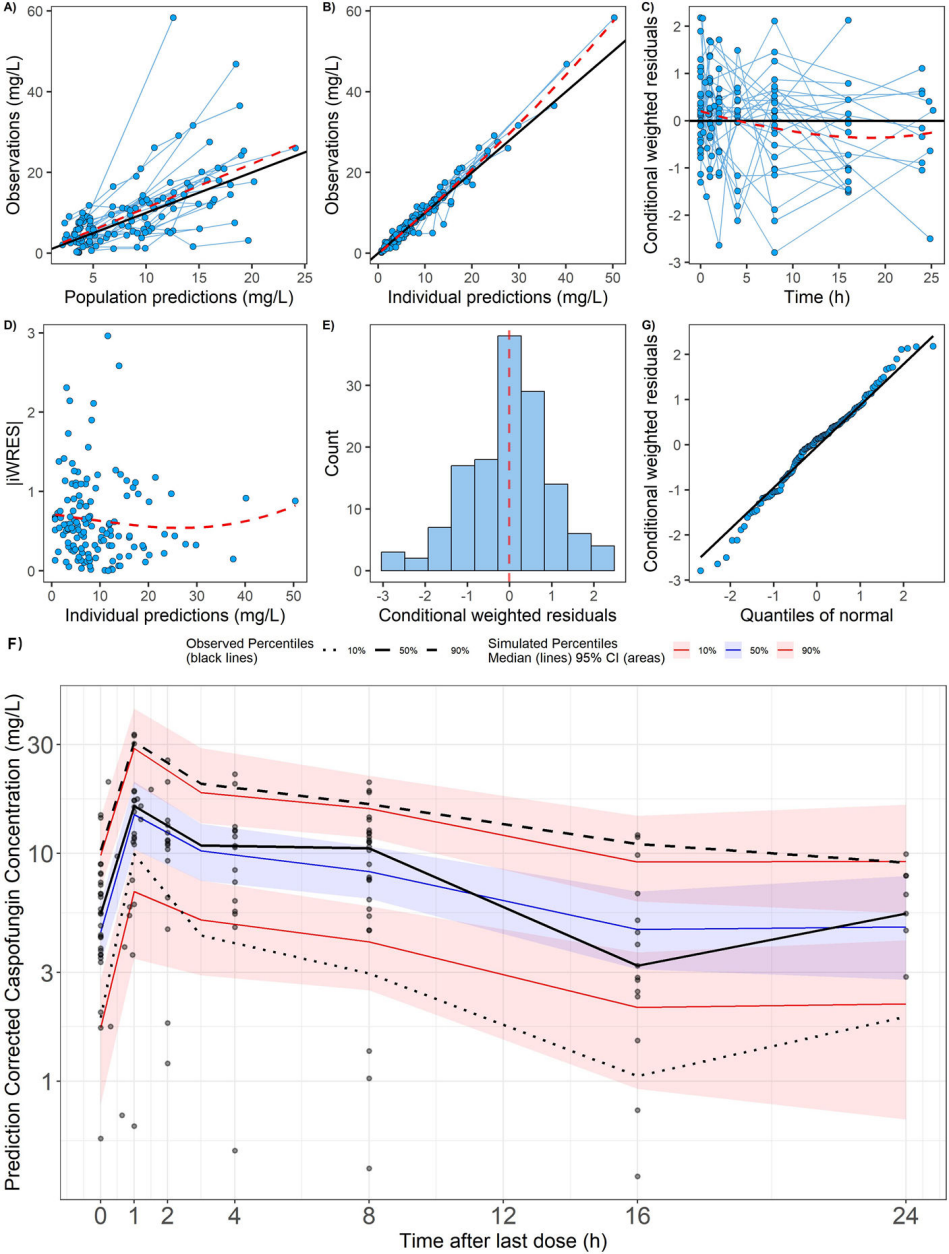
Evaluation of the final caspofungin population pharmacokinetic model. (**A–E**) Goodness of fit (Gof) of final model; (**F**) prediction corrected visual predictive check (pcVPC) of caspofungin concentrations.

**Fig 2:**
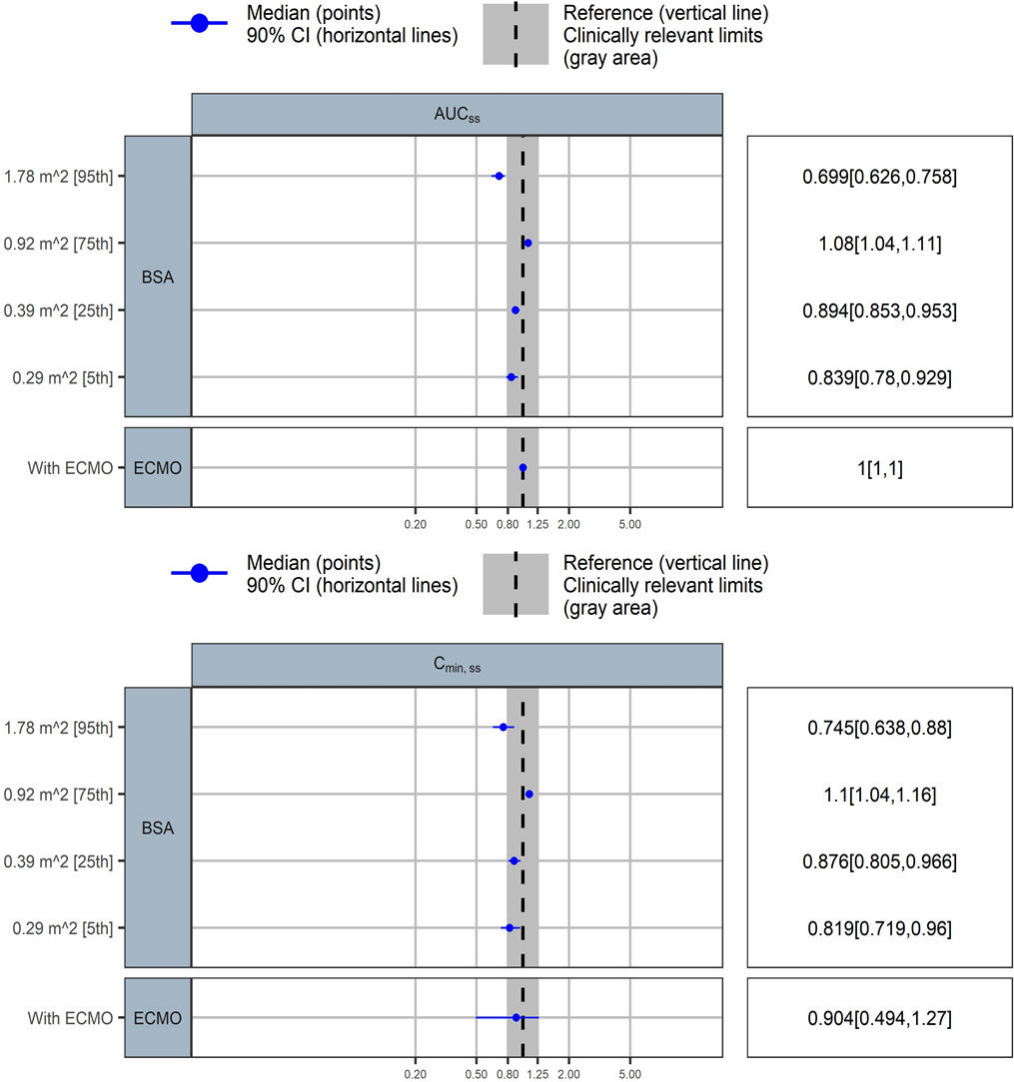
Covariate effects on steady state AUC_ss,24h_ and C_min,ss_ under the standard caspofungin dosing regimen. The blue circles represent the point estimates of covariate effect relative to the reference (typical patient without ECMO support: BSA 0.66 m^2^), and the associated horizontal lines are the 90% confidence intervals. The shaded area ranges from 0.8 to 1.25. Within this area, the covariate effect is not clinically relevant.

All PK parameters were precisely estimated. Final parameter estimates were close to the bootstrap medians and within the 95% confidence intervals, indicating model robustness (Table 2). GoF result and VPC plot (Fig. 1) further confirmed that the final PopPK model effectively represents caspofungin’s PK behavior in the studied population. A BSA-based dosing strategy effectively accounts for the minimal variability observed in AUC_ss,24h_ within the 5th to 75th percentile range (0.29–0.92 m²) (Fig. 2). However, in children with a BSA exceeding 1 m^2^, particularly among those with larger BSA, the AUC_ss,24h_ tended to decrease. While ECMO treatment did not significantly influence AUC_ss,24h_, its impact on the C_min,ss_ fell outside the predefined equivalence range.

### Dosing regimen simulations

**Fig 3:**
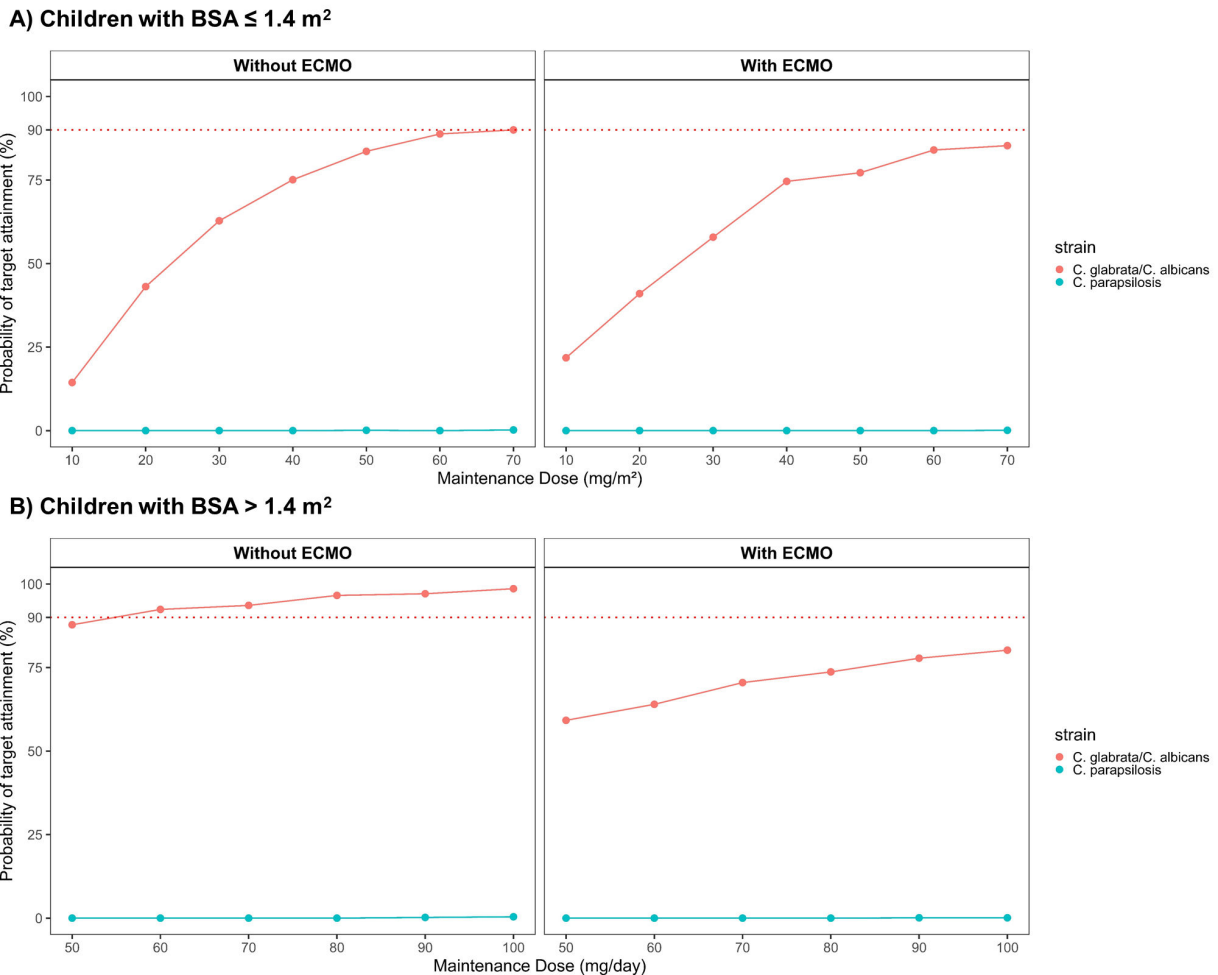
Simulated caspofungin probability of target attainment (PTA) of different maintenance doses using C_min,ss_ as the target; (**A**) PTA for children with BSA ≤ 1.4 m^2^; (**B**) PTA for children with BSA > 1.4 m^2^.

**Fig 4:**
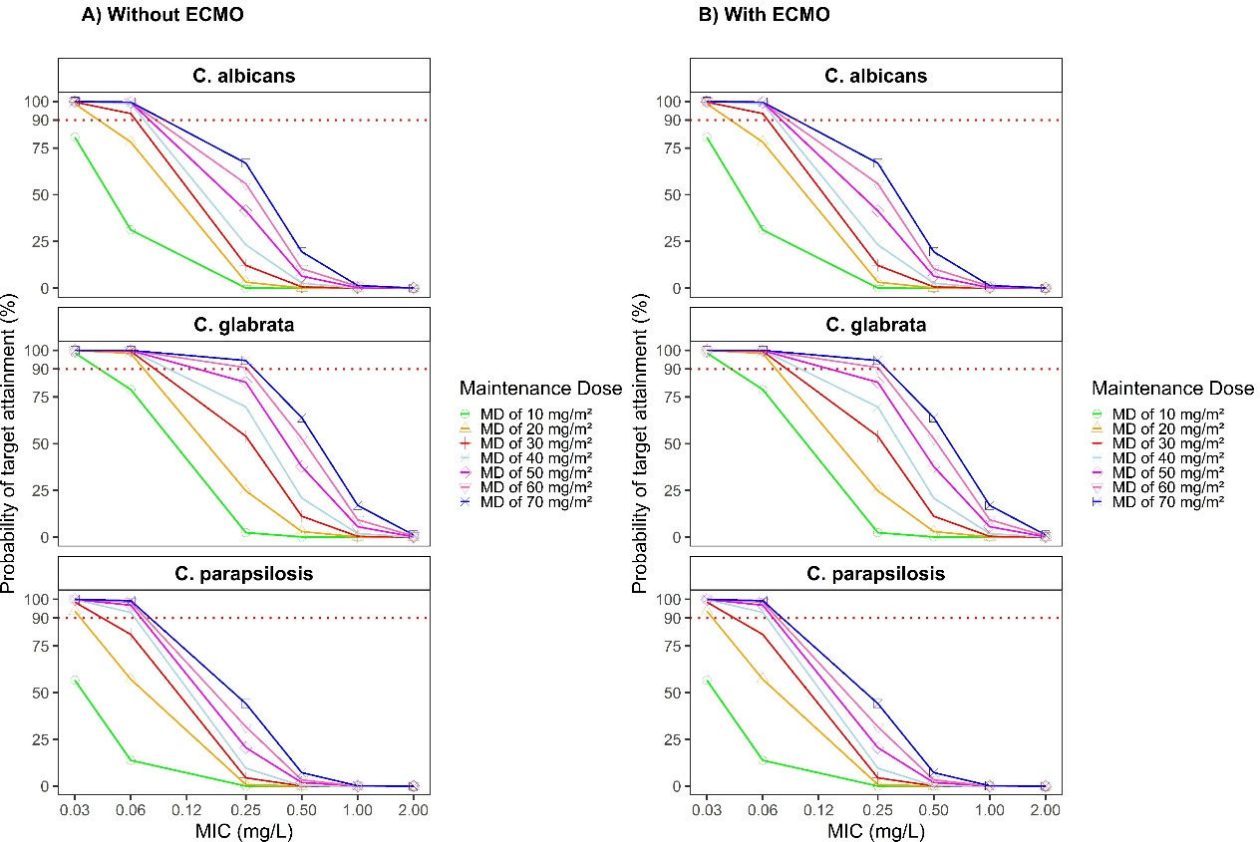
Simulated caspofungin probability of target attainment (PTA) of different maintenance doses using tAUC_ss,24h_/MIC as the target in children with BSA ≤ 1.4 m^2^; (**A**) PTA for children without ECMO support; (**B**) PTA for children with ECMO support.

**Fig 5:**
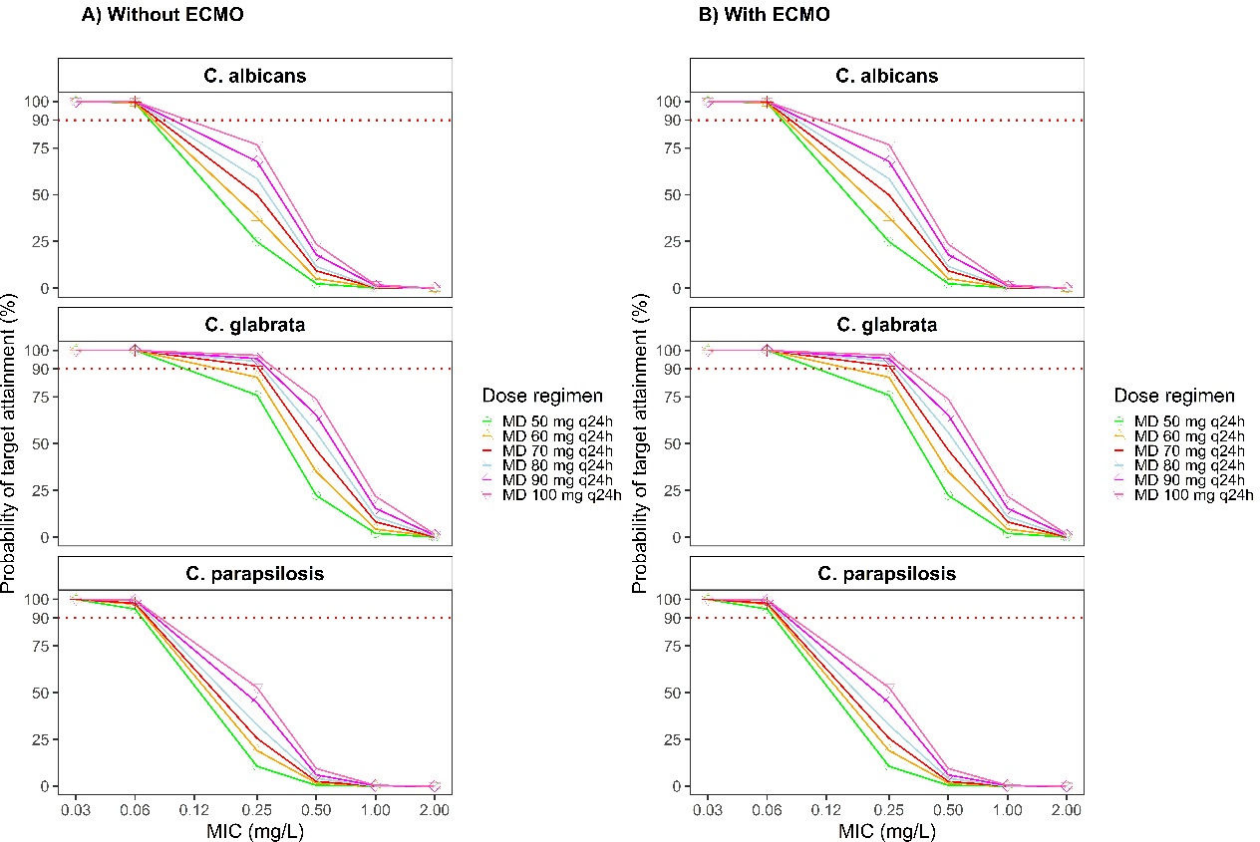
Simulated caspofungin probability of target attainment (PTA) of different maintenance doses using tAUC_ss,24h_/MIC as the target in children with BSA > 1.4 m^2^; (**A**) PTA for children without ECMO support; (**B**) PTA for children with ECMO support.

Figures 3 to 5 illustrated the PTA results for various caspofungin MD regimens. Overall, the observed differences between children receiving ECMO support and those not receiving ECMO support vary depending on the target used. While using C_min,ss_ as the PK/PD target (Fig. 3), PTA differed between pediatric patients with and without ECMO support. In non-ECMO children with BSA ≤ 1.4 m^2^, a maintenance regimen of 70 mg/m² (capped at 70 mg once daily) was sufficient to achieve a PTA exceeding 90%. For non-ECMO children with BSA > 1.4 m², a fixed dose of 60 mg/day was adequate to reach the same PTA threshold. In contrast, among patients receiving ECMO support, none of the simulated dosing strategies were able to achieve a PTA > 90%.

When using tAUC_ss,24h_/MIC as the PK/PD target, the PTA findings showed no apparent differences between pediatric patients with and without ECMO support. Among patients with a BSA ≤ 1.4 m^2^ (Fig. 4), MDs of at least 30 mg/m^2^/day achieved ≥ 90% PTA against *C. albicans* (MIC₉₀ = 0.06 mg/L) and *C. glabrata* (MIC₉₀ = 0.06 mg/L). However, none of the simulated dosing regimens were able to reach the PTA target against *C. parapsilosis* (MIC₉₀ = 1 mg/L). In patients with BSA > 1.4 m² (Fig. 5), a maintenance dose of at least 50 mg/day provided a PTA ≥ 90% against *C. glabrata* (MIC₉₀ = 0.06 mg/L) and *C. parapsilosis* (MIC₉₀ = 0.03 mg/L). In contrast, achieving the same PTA threshold for *C. albicans* (MIC₉₀ = 0.06 mg/L) required a higher maintenance dose. Importantly, once the MIC of the fungal pathogen exceeds these critical breakpoints, even considerable dose escalation of caspofungin fails to achieve the desired 90% PTA.

## DISCUSSION

In this study, we provided the prospective study to quantitatively characterize the pharmacokinetics of caspofungin in Chinese pediatric PICU patients, including those receiving ECMO support. A BSA cutoff of 1.4 m² was identified, above which a fixed maintenance dosing regimen was appropriate. Using the established PK/PD index tAUC_ss,24h_/MIC, ECMO use was found to have no significant effect on dosing requirements.

In this study, we implemented multiple strategies to address the challenges of sample collection in pediatric patients (13, 17, 23). First, we applied optimal sampling design. While reducing the number of samples is the most direct way to minimize burden, it must be carefully balanced against the risk of losing critical PK information. To achieve this balance, we employed a sparse sampling approach guided by optimal design theory, which enabled us to identify the most informative time points for sample collection (24). This strategy allowed us to minimize the number of blood draws while still supporting robust PK analysis through population pharmacokinetic modeling. Importantly, the use of optimal design also facilitated clearer communication during the consent process. By being able to explain and justify the limited number and timing of samples, we were better equipped to reassure caregivers and patients about the study’s safety and scientific validity. This transparency helped reduce apprehension and supported informed decision-making. In addition, recognizing the practical challenges inherent in pediatric studies, our team included both pediatric clinicians and pharmacometricians, who worked closely to ensure that sample collection was feasible, safe, and integrated into routine clinical workflows. This collaborative and patient-centered approach helped build trust and contributed to successful recruitment and retention.

Our results are consistent with three published pediatric PopPK studies that also employed a two-compartment model (25–27). In contrast, one other pediatric study employed a one-compartment model, probably due to its sparse PK data (48 subjects and 139 observations) (28). Compared with the published PK estimates (BSA-normalized clearance, median [range]: 0.208 L/h/m^2^ [0.126–0.21 L/h/m^2^], BSA-normalized volume of distribution (V_1_), median [range]: 2.19 L/m^2^ [1.72–2.62 L/m^2^]), our study showed comparatively higher values of BSA-normalized CL (0.248 L/h/m^2^) and V_1_ (2.81 L/m^2^). The broader BSA range in our study population, which covers very small infants and older children, may partly explain the increased BSA-normalized CL. Additionally, the unique conditions of the critically ill pediatric patients, such as liver insufficiency, hypoproteinemia, and ECMO support, may alter protein binding and blood flow, potentially affecting the drug disposition. Increased vascular permeability and intravenous fluid resuscitation in these critically ill patients may also contribute to elevated V_1_ (29, 30). Despite differences in CL and V_1_, the elimination rate constant (k_e_) in this study (0.088 /h) was generally consistent with those reported in the other four pediatric studies (median: 0.0875 /h; range: 0.074–0.101 /h), supporting the accuracy of the final PopPK model in predicting steady-state exposure.

Previous studies involving pediatric populations typically included body size on PK parameters. Among them, three studies (25, 26, 28) included BSA as a significant covariate on CL and V_1_ while one study (27) emphasized the effect of body weight. In our study, allometric BSA-based allometric scaling yielded lower OFV and AIC compared with weight, LBW, and FFM. The estimated BSA exponent for CL varied considerably across studies, likely reflecting differences in study populations. Du et al. reported the highest exponent of CL (1.98) in adolescents aged 12 to 16.7 years, suggesting a near-quadratic relationship between BSA and CL, possibly due to a narrow age and relatively high BSA range (1.1–2.0 m^2^). In contrast, the BSA exponent for CL reported by Yang et al. (1.3 L/h/m^2^) and Niu et al. (0.89 L/h/m^2^) was lower than that observed in adolescents but higher than in the current study. This discrepancy may be attributable to the underrepresentation of neonates and infants with low BSA values in their cohorts (Yang et al.: 0.54–1.39 m^2^; Niu et al.: 0.40–1.50 m^2^), which could have led to an overestimation of the BSA exponent for CL. Notably, our current study identified a fixed BSA-exponent of 0.66, consistent with allometric theory, and demonstrated stability across a wider PICU cohort (0.33–16 years, BSA 0.29–1.89 m²). Other factors, including AST and ALT levels, did not appear to influence the PK of caspofungin.

In our study, ECMO was associated with a marked 18.2-fold increase in V_1_, suggesting altered drug disposition in this population. The increased V_1_ observed in our study might reflect enhanced drug sequestration in the ECMO circuit, increased capillary permeability, or altered plasma protein binding in pediatric patients. Bayesian posterior estimates further revealed a lower median although not statistically significant AUCₛₛ,_24h_ in patients receiving ECMO treatment (ECMO group [*n* = 4, median: 117.08 h*mg/L, range: 17.20–176.04 h*mg/L] vs non-ECMO group [*n* = 25, median: 193.63 h*mg/L, range: 53.18–380.95 h*mg/L], *P* = 0.06664). However, it is important to note that the *P*-value was very close to the conventional threshold of 0.05, indicating a marginal difference that approached statistical significance. Moreover, the sample sizes between the ECMO and non-ECMO groups were notably imbalanced. Therefore, further investigations with larger and more balanced cohorts are warranted to robustly assess the impact of ECMO on caspofungin PKs in PICU patients.

When C_min,ss_ was adopted as the PK/PD target, a marked difference in PTA was observed between patients with and without ECMO support. Even with increased MDs, ECMO patients failed to achieve the 90% PTA threshold, which may be attributed to the fact that ECMO primarily influences V_1_, given that AUC equals dose divided by clearance. In our study, ECMO primarily influenced V_1_, which may explain the observed reduction in trough concentrations without significant changes in overall exposure. It is noteworthy that although standard dosing regimens were sufficient to reach the target tAUC_ss,24h_/MIC, they did not adequately maintain C_min,ss_ above the threshold. This phenomenon was particularly evident in patients undergoing ECMO but was also observed in non-ECMO populations, consistent with the findings reported by Du et al. (25).

The inconsistency between tAUC_ss,24h_ and C_min,ss_ based PTA results likely reflects the distinct pharmacodynamic implications of these indices, tAUC_ss,24h_ captures overall drug exposure, whereas C_min,ss_ emphasizes sustained concentrations above a critical threshold. While the tAUC_ss,24h_/MIC is a reasonable and widely accepted metric (9, 31–33), C_min,ss_ may still have practical utility (25), particularly in settings where AUC estimation is not feasible. However, both targets are primarily derived from preclinical models and *in vitro* data and have not yet been established through clinical studies (21). The discrepancies found in our PICU patients, where PTA varied depending on the PK/PD metric used, highlight the need for careful evaluation of both exposure parameters in complex clinical scenarios. Therefore, both indices should be interpreted in the context of the drug’s pharmacological properties and clinical outcomes. AUC_ss,24h_/MIC is a well-established and pharmacodynamically robust efficacy marker, and it should be prioritized as the primary PK/PD target for dose optimization, particularly in clinical settings where precise exposure–response relationships are critical.

Our study has several limitations. First, the lack of PK sampling after the first dose precluded the ability to make evidence-based recommendations regarding the loading dose. Second, the physiological condition of PICU patients may fluctuate over time, and single-day sampling after reaching steady state may not adequately capture time-varying covariates and inter-occasion variability. Further multi-day research is necessary to address this knowledge gap to better assess the PK characteristics of caspofungin in PICU pediatric populations. Additionally, our study focused solely on total concentrations. Since caspofungin has a high protein binding rate, and studies have indicated that albumin levels can influence its pharmacokinetics (34), future research should also consider unbound concentrations of caspofungin for a more comprehensive analysis. Finally, although only four patients received ECMO treatment in this study, their data still provided valuable preliminary insights into antifungal pharmacokinetics under ECMO support. Therefore, these results were included in our analysis. Nevertheless, given the small sample size, the related conclusions should be interpreted with caution, and future studies with larger cohorts of ECMO patients are warranted to confirm and extend these findings.

### Conclusion

The current BSA-based dosing regimen is appropriate for PICU patients with a BSA ≤ 1.4 m², while a fixed-dose regimen appears more suitable for those with a BSA > 1.4 m². Additionally, PICU patients undergoing ECMO therapy exhibit altered pharmacokinetics, and further studies are needed to clarify whether dose adjustments are required to achieve optimal caspofungin exposure.

## Data Availability

The data generated during and/or analyzed during the current study are available from A/Prof Yixue Wang or A/Prof Xiao Zhu on reasonable request.

## SUPPLEMENTAL MATERIAL

Supplemental material aac.01277-25-s0001.docx Fig. S1 to S5; Tables S1 to S6.

ASM does not own the copyrights to Supplemental Material that may be linked to, or accessed through, an article. The authors have granted ASM a non-exclusive, world-wide license to publish the Supplemental Material files. Please contact the corresponding author directly for reuse.
