# Supplementary material for: Population pharmacokinetics of caspofungin in critically ill Chinese children: a prospective observational study
Source: Antimicrob Agents Chemother. 2025 Dec 30;70(2):e01277-25. doi: 10.1128/aac.01277-25 (PMC12888871; doi:10.1128/aac.01277-25)
Supplement: Supplemental material — Fig. S1 to S5; Tables S1 to S6. [file aac.01277-25-s0001.docx]

**Optimal sampling design for caspofungin**

**Method**

During the optimized sampling process, explicit constraint conditions were defined. In the second phase of our study, approximately 40 participants were to be recruited to receive a daily 1-hour intravenous infusion of caspofungin, with a BSA-based dosing strategy. Sampling was planned to take place within one single day after reaching steady-state levels. Given that the study subjects were pediatric patients, we aimed to minimize the number of sampling points.

Design software tools, including $DESIGN option in NONMEM and PopED (version 0.6.0, R package), were utilized to explore design elements such as sampling size, and sampling times. These tools evaluated uncertainties in PK parameter estimates based on the current sampling scheme and population pharmacokinetic (PPK) model. The designs were evaluated to ensure the precise estimation of typical PK parameters, defined as a relative standard error (RSE) of less than 50%.

Sensitive analysis was performed on the continuous covariates in the final PPK model, exploring the optimization results at 5^th^ percentile and 95^th^ percentile of these covariates to assess their influence on the final optimization outcomes. Sampling window analysis were also performed to explore a more practical design by looking at the loss of efficiency when sample times are distributed in a way that aligns with real-world conditions around the optimal points. The final sampling design was further validated through stochastic simulation and estimation (SSE, n=1000).

Evaluation of sampling scheme transferability was performed using four PPK models from different pediatrics studies. This evaluation assessed the feasibility of applying the scheme to other non-ICU pediatric populations.

**Result**

The initial intensive sampling design, which consisted of sampling intervals of 120, 121, 122, 124, 128, and 136 hours, was evaluated through $DESIGN in NONMEM (Table S1). The evaluation result from $DESIGN in NONMEM showed that RSEs of typical parameters were: 12% for CL, 6% for V_1_, 12% for Q, and 43% for V_2_. This suggested that PK parameters could be estimated with reasonable precision based on the initial sampling strategy. Additionally, these RSE results closely matched those obtained from SSE (considered as the reference, Table S1), further validating the initial sampling time points.

We explored various designs with different numbers of samples per patient and found that at least four sampling points were required to accurately characterize the PK behavior of caspofungin. As shown in Table 3, although five sampling time points could ensure the precise estimation of PK parameters, but the third time point (127.16h) and the fourth time point (127.31h) was very close and could potentially be combined into one single sampling point to reduce the discomfort of sampling for children. Reducing the number of sampling points to three resulted in a larger RSE for the typical parameters: RSE of V_2_ > 50%. Four sampling points per subject were sufficient for precise parameter estimation, with RSEs remaining below 45%. The optimized sampling time points were identified as 119 hours, 121 hours, 126.5 hours, and 144 hours after the first dose using $DESIGN.

Sensitivity analysis (Figure S3) showed minimal impact of weight and AST levels on the optimized design, validating its robustness across a range of conditions (Figure S3). For weight, at the 5^th^ and 95^th^ percentiles, the third sampling point shifted slightly from 124.97 hours to 127.97 hours, with no significant deviation from the results based on median weight. Similarly, AST had minimal impact, with the third sampling point varying between 125.09 hours and 128.01 hours. Sampling within specific time windows (119-120 hours, 120.5-121.5 hours, 126-127 hours, and 143-144 hours) proved to be highly efficient, preserving over 95% of the information compared to fixed D-optimal time points (Figure S4A). The SSE results further confirmed that PK parameters and inter-individual variability could be estimated with greater precision. The RSEs of typical parameters and inter-individual variability parameters were less than 50% and 35%, respectively, with relative biases not exceeding ±15% (Figure S4B, Figure S4C).

The evaluation of sampling scheme transferability was shown in Figure S5. Despite the inconsistency in the study populations, the RSE median of the typical pharmacokinetic parameters did not exceed 60% within the current sampling windows. Although the median of bias of typical pharmacokinetic parameters were within the range of ±20% (Figure S5), that of IIV exceeded ±80% in two studies. This suggested that the current sampling scheme may not adequately estimate IIV when applied to other patient populations.

**
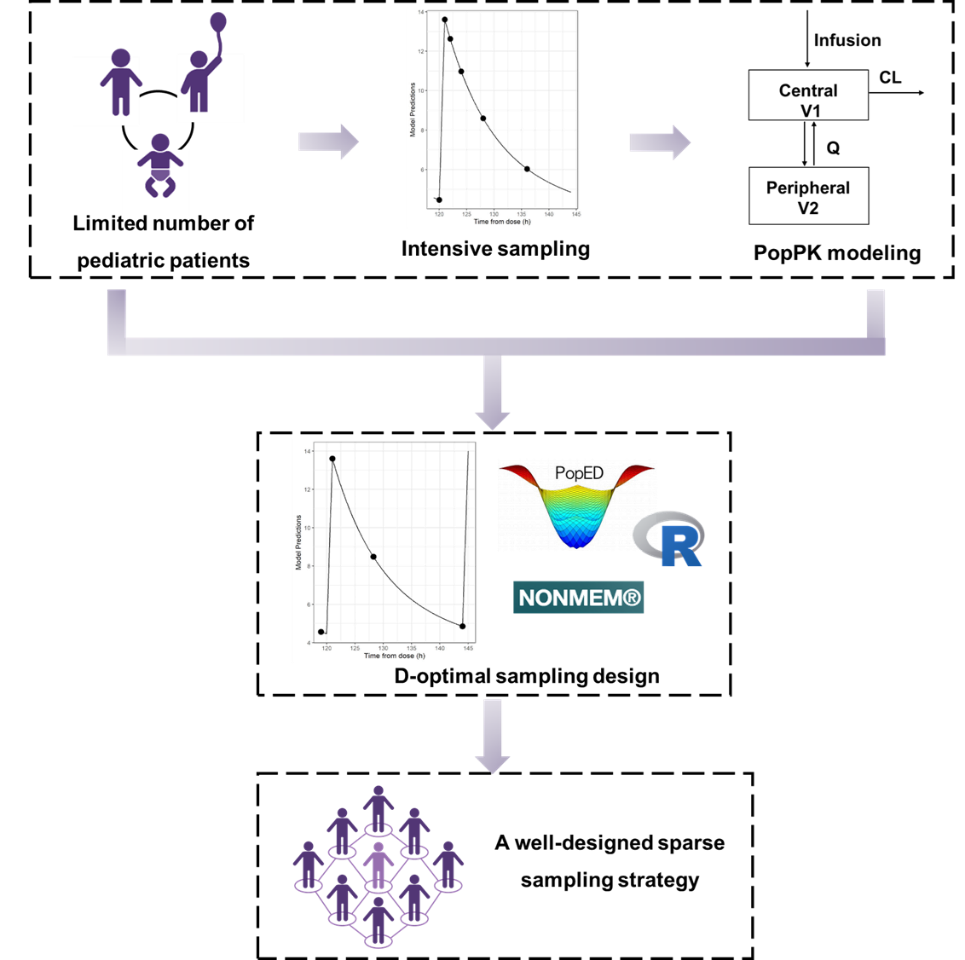
**

**Figure S1**. Study flow diagram. In the first stage of the study, a population pharmacokinetic model was developed based on intensive sampling data collected from the prospective clinical study conducted among pediatrics with a small sample size in ICU. Secondly, design software tools, including $DESIGN in NONMEM and PopED, were utilized to optimize sampling design. During the third phase in our study, the optimized sampling scheme would be applied to a larger-scale study.

**
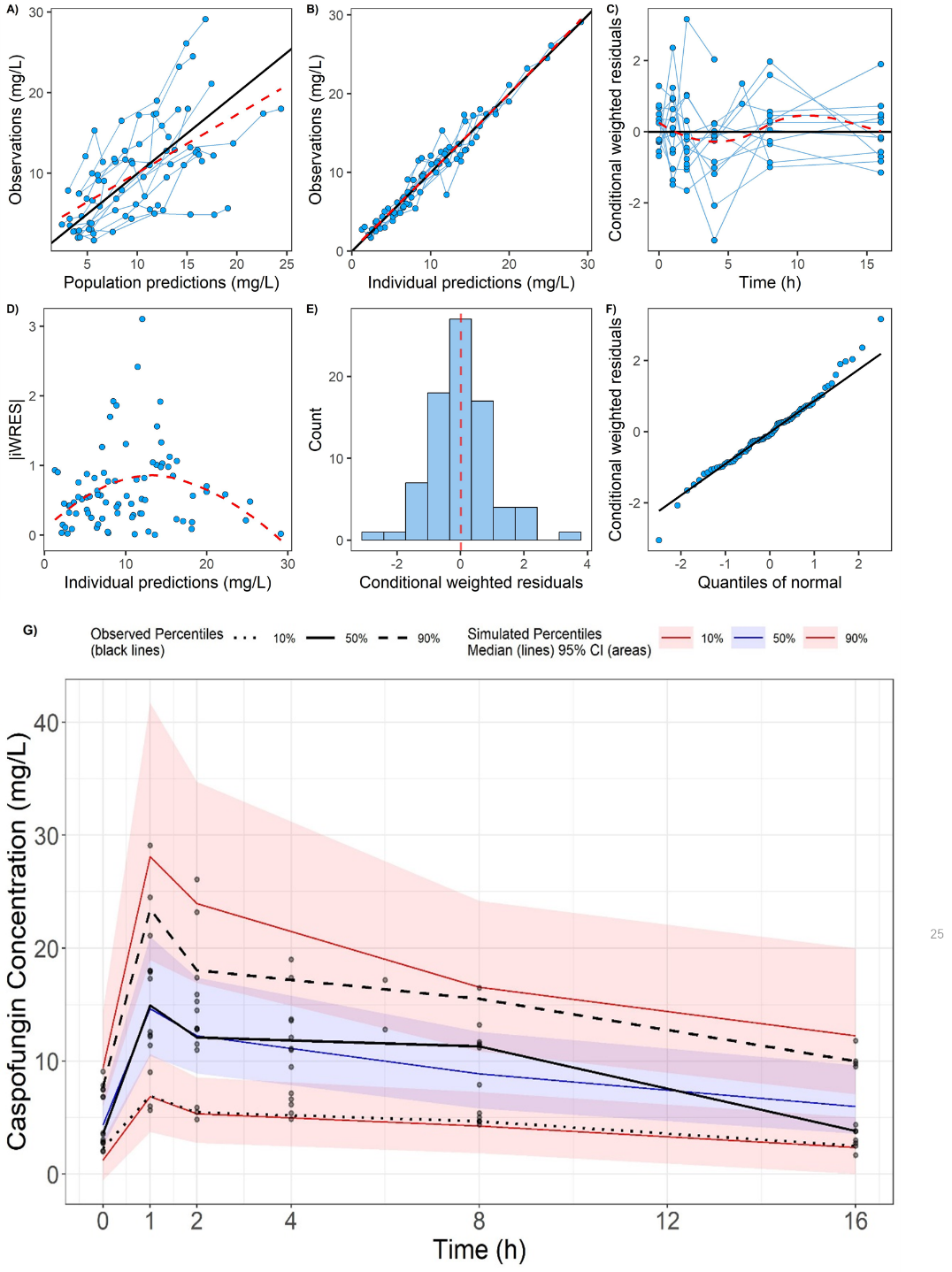
**

**Figure S2**. Model internal evaluation of the final population pharmacokinetic model in steady state.

A) – F) The goodness-of-fit plot of caspofungin concentrations. G) Visual predictive check plot. Black lines represent the 10% (dashed), 50% (solid), and 90% (dashed) percentiles of the observed data. Shaded areas represent 95% confidence intervals of the 10% (red), 50% (blue), and 90% (red) percentiles of the model prediction.


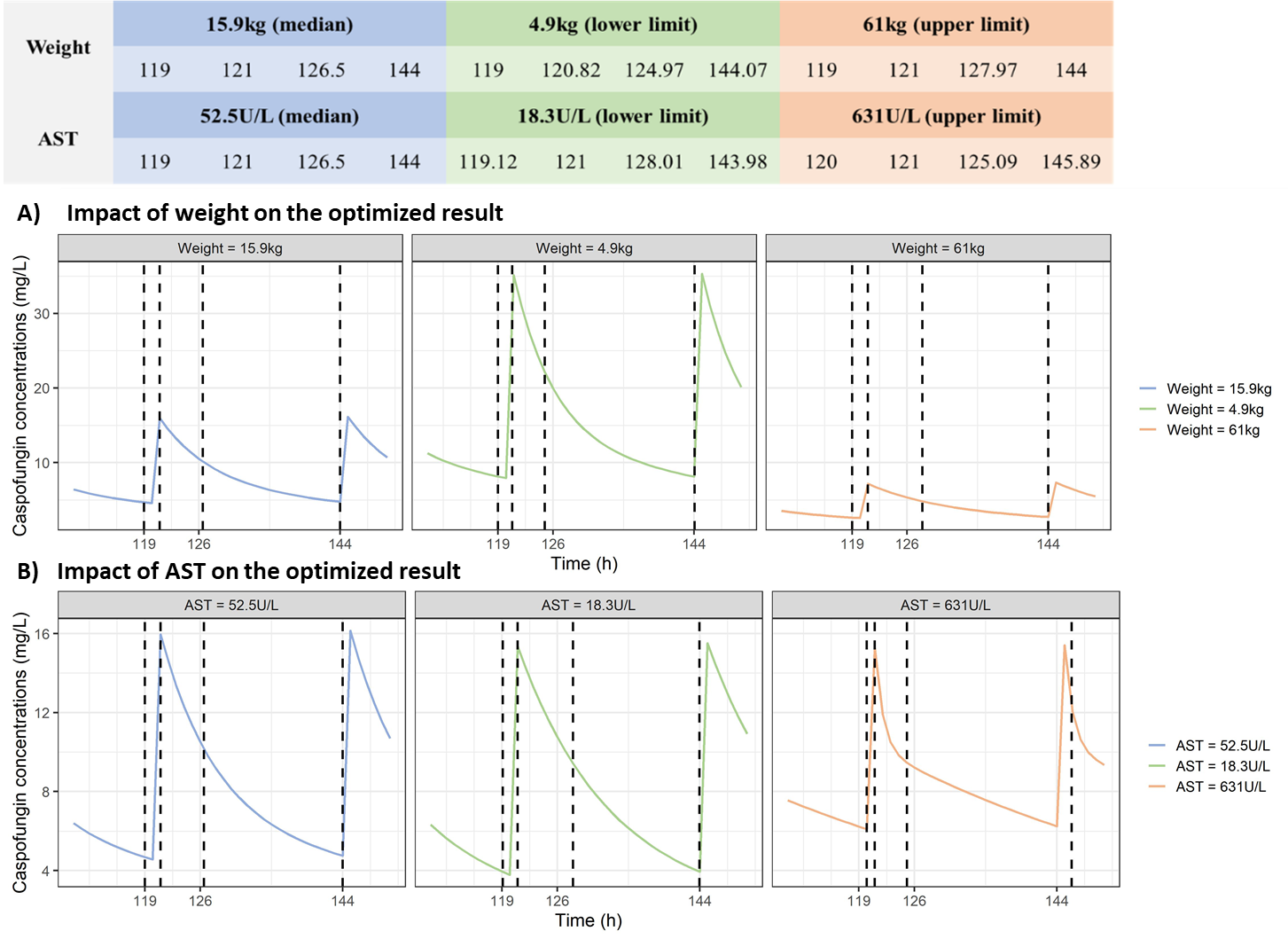


**Figure S3.** Sensitivity analysis of different covariates effects on the optimized result,along with the typical pharmacokinetic profile. A) Optimized results using $DESIGN in NONMEM based on different weight values; B) Optimized results using $DESIGN in NONMEM based on different AST values. Weight = 4.9kg, 15.9kg and 61kg are the 5^th^, 50^th^ and 95^th^ percentiles of included patients; AST = 18.3U/L, 52.5U/L and 631U/L are the 5^th^, 50^th^ and 95^th^ percentiles of included patients.

**
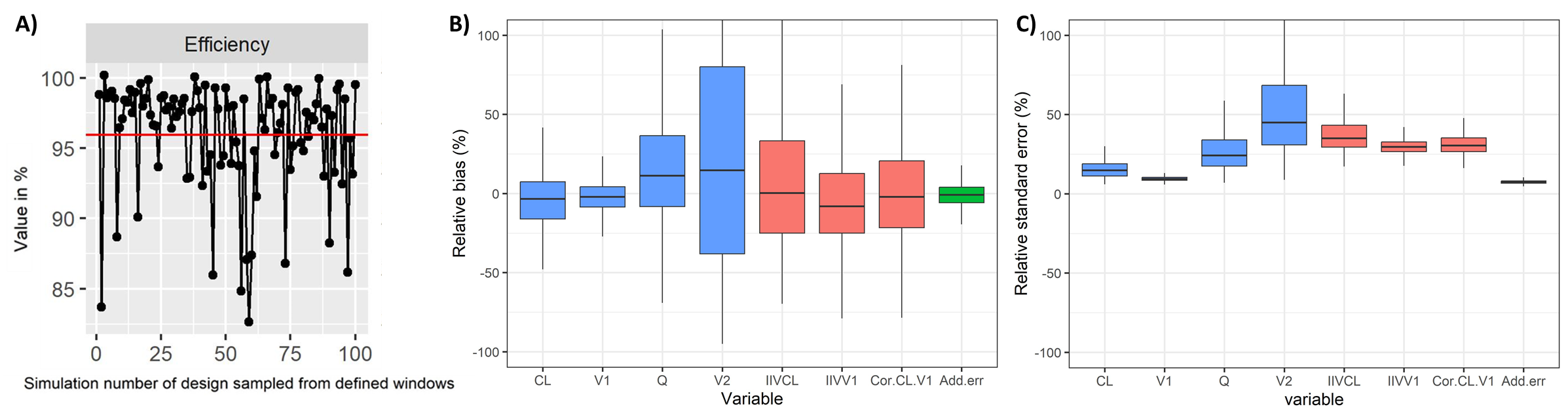
**

**Figure S4.** The performance of the proposed sampling windows (119-120 h, 120.5-121.5 h, 126-127 h, and 143-144 h). A) The efficiency plot of sampling windows; B) Relative bias (%) of PPK parameters estimation; C) Relative standard error (%) of PPK parameters estimation. Box lines: upper quartile, lower quartile, and median value; Variables: CL clearance, V_1_, volume of distribution of the central compartment, Q, atrioventricular exchange rate, V_2_, volume of distribution of the peripheral compartment, IIV inter-individual variability; Cor.CL.V_1_ correlation between CL and V_1_.

**
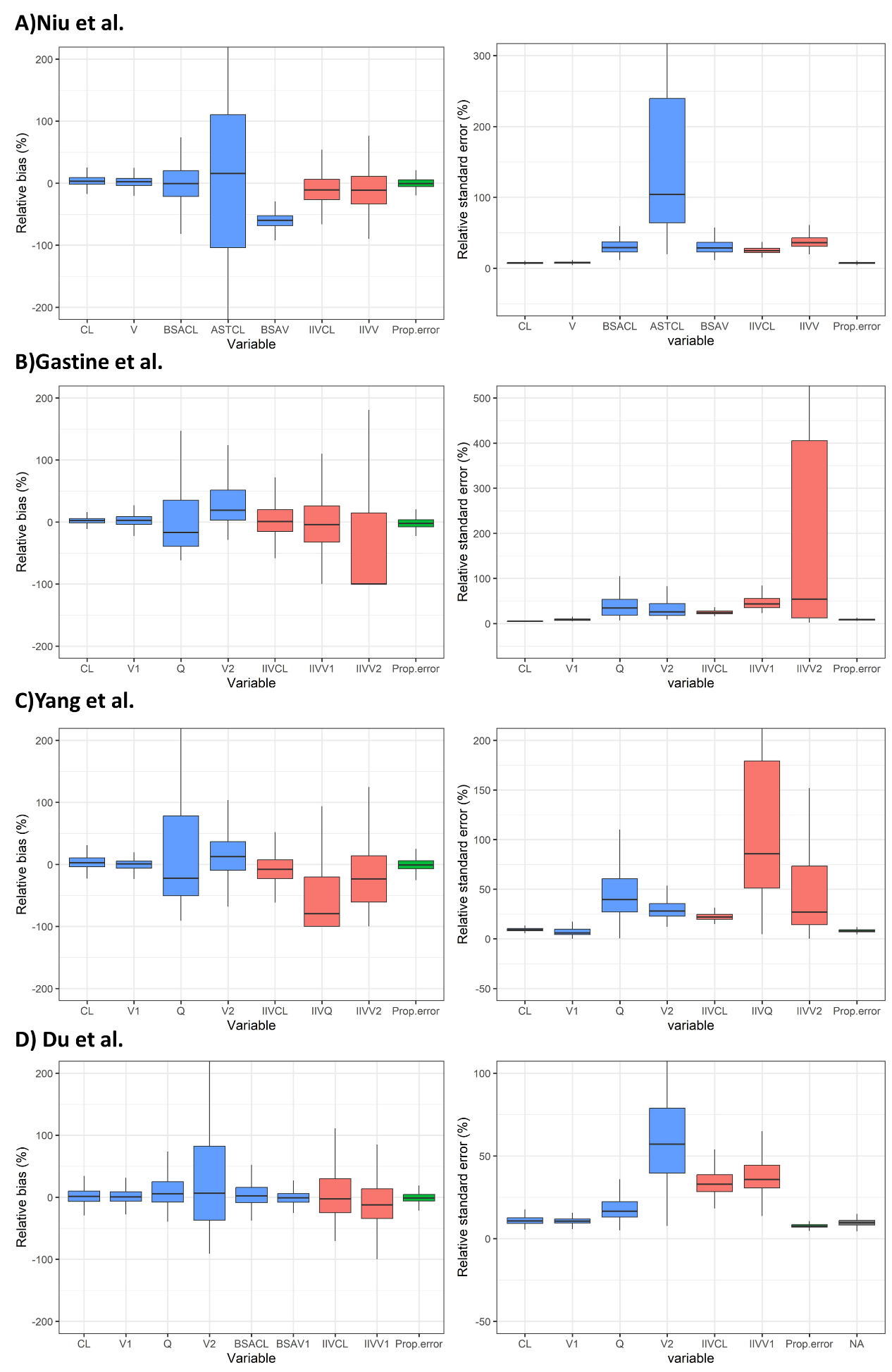
Figure S5.** Stochastic simulation and estimation (SSE) of the proposed sampling windows based on four published population pharmacokinetics models of caspofungin.

**Table S1.** Demographic and laboratory information of patients

| **Characteristics** | **Median [min, max] (n=14)** |
| --- | --- |
| **Demographic information** | |
| ECMO (Yes/No) | 3/11 [21.4%/78.6%] |
| Age (years) | 4.63 [0.330, 16.0] |
| Sex (Male/Female) | 5/9 [35.7%/64.3%] |
| Weight (kg) | 15.9 [4.90, 64.0] |
| Height (cm) | 97.5 [54.0, 160] |
| Body Surface Area (m^2^) | 0.655 [0.286, 1.69] |
| Body Mass Index (kg/m²) | 18.01 [9.82 – 25.64] |
| **Laboratory information** | |
| White Blood Cell (10^9^/L) | 6.37 [1.56, 22.5] |
| Red Blood Cell (10^12^/L) | 2.67 [1.88, 5.25] |
| Hemoglobin (g/L) | 77.0 [51.0, 153] |
| Platelet (10^9^/L) | 134 [18.0, 434] |
| Albumin (g/L) | 36.0 [24.7, 49.9] |
| Total Protein (g/L) | 61.6 [36.8, 75.4] |
| Alanine Aminotransferase (IU/L) | 17.0 [3.51, 223] |
| Aspartate Aminotransferase (IU/L) | 52.5 [18.3, 631] |
| Total Bilirubin (μmol/L) | 26.6 [2.10, 357] |
| Direct Bilirubin (μmol/L) | 13.1 [1.10, 228] |
| Serum Creatinine (μmol/L) | 27.7 [14.0, 223] |
| Uric Acid (μmol/L) | 177 [87.0, 736] |

**Table S2.** Parameter estimates of the final caspofungin population pharmacokinetic model

| **Fixed Parameters** | **Final Estimates** | **RSE^a^** | **SIR median**  **[95% CI^c^]** |
| --- | --- | --- | --- |
| CL, L/h | 0.477 | 18% | 0.494 [0.372 – 0.625] |
| V_1_, L | 11.8 | 15% | 12.18 [9.66 – 15.17] |
| Q, L/h | 0.512 | 23% | 0.55 [0.32 – 0.81] |
| V_2_, L | 19.2 | 41% | 19.36 [9.98 – 28.62] |
| *The effect of* ***weight*** *on CL* | 0.75 FIX | / | / |
| *The effect of* ***weight*** *on V_1_* | 1 FIX | / | / |
| *The effect of* ***weight*** *on Q* | 0.75 FIX | / | / |
| *The effect of* ***weight*** *on V_2_* | 1 FIX | / | / |
| *The effect of* ***AST*** *on Q* | 0.898 | 16% | 0.875 [0.655 – 1.075] |
| **Residual error** | **Estimates** | **RSE [SHR]^b^** | **/** |
| Add. err, mg/L | 1.58 | 16% [14%] | 1.62 [1.32 – 2.02] |
| **Inter-individual variability(%CV)** | **Estimates** | **RSE [SHR]^b^** | **/** |
| CL | 64.4% | 15.8% [0%] | 69.79% [50.27% - 86.46%] |
| V_1_ | 50.3% | 25.1% [2%] | 55.41% [36.28% - 74.56%] |
| Cor_CL-V1_ | 82.4% | 34.06% | 79.44% [70.02% - 84.44%] |
| ***CL = 0.477 x (weight/70)^0.75^; V_1_ = 11.8 x (weight/70)^1^;***  ***Q = 0.512 x (weight//70)^0.75^ x (AST/52.5)^0.898^; V_2_ = 19.2 x (weight/70)^1^***  RSE: relative standard error; b. SHR: shrinkage; c. CI: confidence interval; SIR: sampling importance resampling. | | | |
| CL clearance; V_1_, volume of distribution of the central compartment; Q, atrioventricular exchange rate; V_2_, volume of distribution of the peripheral compartment; AST, Aspartate aminotransferase | | | |

**Table S3.** Results of the optimal study design process

| **Item** | **Optimization 1** | **Optimization 2** | **Optimization 3** |  |
| --- | --- | --- | --- | --- |
| %RSE(CL) | 11% | 12% | 13% |  |
| %RSE(V1) | 6% | 6% | 6% |  |
| %RSE(Q) | 10% | 12% | 14% |  |
| %RSE(V2) | 42% | 43% | 59% |  |
| %RSE(Add.err) | 13% | 16% | 22% |  |
| %RSE(var(CL)) | 12% | 12% | 13% |  |
| %RSE(var(V1)) | 13% | 14% | 15% |  |
| %RSE(var  (Cor_CL_V1)) | 13% | 13% | 13% |  |
| Sampling strategy | | | |  |
| Sample Time 1 (h) | 119 | 119 | 120 |  |
| Sample Time 2 (h) | 121 | 121 | 127.63 |  |
| Sample Time 3 (h) | 127.16 | 126.5 | 145 |  |
| Sample Time 4 (h) | 127.31 | 144 | / |  |
| Sample Time 5 (h) | 144 | / | / |  |

**Table S4.** Results of the optimal study design and SSE of the final sampling windows

| **Item** | **SSE** | **$DESIGN in NONMEM** |
| --- | --- | --- |
| %RSE(CL) | 14.75% | 12% |
| %RSE(V1) | 9.43% | 6% |
| %RSE(Q) | 24.12% | 12% |
| %RSE(V2) | 45.02% | 43% |
| %RSE(Add.err) | 7.33% | 16% |
| %RSE(var(CL)) | 35.02% | 12% |
| %RSE(var(V1)) | 29.61% | 14% |
| %RSE(var  (Cor_CL_V1)) | 30.45% | 13% |
| Sample Time 1 (h) | 119-120 | 119 |
| Sample Time 2 (h) | 120.5-121.5 | 121 |
| Sample Time 3 (h) | 126-127 | 126.5 |
| Sample Time 4 (h) | 143-144 | 144 |

**Table S5.** Evaluation of initial sampling design.

| **Item** | **SSE (median)** | **$DESIGN in NONMEM** |
| --- | --- | --- |
| %RSE(CL) | 14.77% | 12% |
| %RSE(V_1_) | 8.21% | 6% |
| %RSE(Q) | 11.25% | 12% |
| %RSE(V_2_) | 34.84% | 43% |
| %RSE(Add.err) | 5.41% | 11% |
| %RSE(var(CL)) | 34.96% | 12% |
| %RSE(var(V_1_)) | 24.10% | 13% |
| %RSE(var  (Cor_CL_V_1_)) | 27.87% | 13% |

RSE: relative standard error; CL clearance; V_1_, volume of distribution of the central compartment; Q, atrioventricular exchange rate; V_2_, volume of distribution of the peripheral compartment; Add.err, addictive error

**Table S6**. Incorporation of body size during base model development

| **Run** | | **Description** | power_CL/Q | power_V_1_/V_2_ | **OFV** | **AIC** | **SC** |
| --- | --- | --- | --- | --- | --- | --- | --- |
| 1 | 2-comp | | / | / | 453.430 | 473.430 | Y |
| 2 | 2-comp + allometric scaling (WT) | | 0.75 FIX | 1 FIX | 438.633 | 456.633 | Y |
| 3 | 2-comp + allometric scaling (LBW) | | 0.75 FIX | 1 FIX | 438.798 | 456.798 | Y |
| 4 | 2-comp + allometric scaling (FFM) | | 0.75 FIX | 1 FIX | 438.535 | 456.535 | Y |
| **5** | **2-comp + allometric scaling (BSA)** | | 0.66 FIX | 1 FIX | **433.889** | **451.889** | **Y** |
| 6 | 2-comp + covariate (WT) | | 0.634 | 0.713 | 436.187 | 458.187 | Y |
| 7 | 2-comp + covariate (LBW) | | 0.624 | 0.683 | 434.292 | 456.292 | N |
| 8 | 2-comp + covariate (FFM) | | 0.643 | 0.726 | 435.502 | 457.502 | Y |
| 9 | 2-comp + covariate (BSA) | | 0.856 | 1.05 | 433.188 | 455.188 | Y |

**Final PPK model for the design time points optimization in NONMEM**

$PROBLEM PK

$INPUT C ID TIME AMT ADDL RATE II EVID MDV DV TSTRAT TMIN TMAX WT AST

$DATA D_cas_optimization.csv IGNORE=C

$SUBROUTINES ADVAN3 TRANS4

$PK

TVCL = THETA(1) * (WT/70)**0.75

CL = TVCL * EXP(ETA(1))

TVV1 = THETA(2) *(WT/70) **1

V1 = TVV1 * EXP(ETA(2))

TVQ = THETA(3) * (WT/70)**0.75 * (AST/52.5)**0.898

Q = TVQ * EXP(ETA(3))

TVV2 = THETA(4) *(WT/70) **1

V2 = TVV2 * EXP(ETA(4))

$ERROR

IPRED=A(1)/V1

Y=IPRED + IPRED*EPS(1) + EPS(2)

$THETA

0.477 ; CL

11.8 ; V1

0.512 ; Q

19.2 ; V2

$OMEGA BLOCK(2)

0.415 ; IIV_CL

0.267 0.253 ; IIV_V1

$OMEGA

0 FIXED ; IIV_Q

0 FIXED ; IIV_V2

$SIGMA

0 FIXED ; Prop.error

1.58 ; Add.error

$DESIGN GROUPSIZE=40 FIMDIAG=2 MAXEVAL=9999 PRINT=20 DESEL=TIME DESELSTRAT=TSTRAT DESELMIN=TMIN DESELMAX=TMAX
